# Patch‐wise brain age longitudinal reliability

**DOI:** 10.1002/hbm.25253

**Published:** 2020-11-18

**Authors:** Iman Beheshti, Olivier Potvin, Simon Duchesne

**Affiliations:** ^1^ Centre de recherche CERVO Québec Canada; ^2^ Département de radiologie et de médecine nucléaire, Faculté de médecine Université Laval Québec Canada

**Keywords:** anatomical MRI, brain age, estimation, longitudinal study, patch‐wise grading, reliability

## Abstract

We recently introduced a patch‐wise technique to estimate brain age from anatomical T1‐weighted magnetic resonance imaging (T1w MRI) data. Here, we sought to assess its longitudinal reliability by leveraging a unique dataset of 99 longitudinal MRI scans from a single, cognitively healthy volunteer acquired over a period of 17 years (aged 29–46 years) at multiple sites. We built a robust patch‐wise brain age estimation framework on the basis of 100 cognitively healthy individuals from the MindBoggle dataset (aged 19–61 years) using the Desikan‐Killiany‐Tourville atlas, then applied the model to the volunteer dataset. The results show a high prediction accuracy on the independent test set (*R*^2^ = .94, mean absolute error of 0.63 years) and no statistically significant difference between manufacturers, suggesting that the patch‐wise technique has high reliability and can be used for longitudinal multi‐centric studies.

## Graphical abstract

We assessed the performance of a patch‐wise technique for brain age estimation in a longitudinal context. We leveraged a unique MRI dataset of 99 T1w MRIs acquired over 17 years in a single volunteer as our test set. The patch‐wise technique demonstrated a high level of reliability for longitudinal brain age estimation studies.

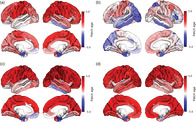

## 1 INTRODUCTION

Brain age estimation has become a research topic of considerable interest in neuroimaging studies, progressively attracting attention from both clinical and engineering communities (Cole, Marioni, Harris, & Deary, 2019). In the last decade, substantial efforts have been devoted toward the development of highly accurate brain age estimation frameworks through modalities as different as anatomical MRI (K. Franke, Ziegler, Kloppel, Gaser, & Alzheimer's Disease Neuroimaging Initiative, 2010), fluorodeoxyglucose positron emission tomography imaging (Goyal et al., 2019), and brain electroencephalogram signals (Al Zoubi et al., 2018).

Brain age estimation techniques based on anatomical MRI can be classified into three approaches: (1) *voxel‐wise techniques*, introduced by Franke and colleagues (K. Franke et al., 2010), that use voxel signal intensities obtained from gray matter (GM), white matter (WM) or a combination of both as dependent variables in the brain age estimation framework. An extensive review of the voxel‐wise technique and its application in neuroimaging studies is presented in (Katja Franke & Gaser, 2019); (2) *region‐wise techniques*, as proposed by Valizadeh, Hanggi, Merillat, and Jancke (2017), employ brain anatomical measures such as those obtained with a segmentation algorithm (e.g., *FreeSurfer* [http://freesurfer.net]) as dependent variables in the brain age estimation framework (Pardoe & Kuzniecky, 2018). These techniques have been used in investigations of brain age not only among healthy individuals, but also for different neurological diseases (Katja Franke & Gaser, 2019); and (3) *patch‐wise grading*, introduced by Beheshti and peers (Beheshti, Gravel, Potvin, Dieumegarde, & Duchesne, 2019) as the most recent approach in the field. It uses image similarity metrics to match test patches to known labels from a library of training set, then weighing and averaging information from the training set (e.g., chronological age) to derive final values (e.g., brain age) on the unseen test image.

Although both voxel‐ and region‐wise techniques have shown acceptable prediction accuracies (i.e., mean absolute error [MAE] ranging from four to 8 years), the patch‐wise technique demonstrated an increased prediction accuracy on an independent test set (MAE < 2 years; Beheshti et al., 2019).

While all three brain estimation paradigms have been widely used in cross‐sectional studies, there have been few investigations of their reliability for longitudinal brain age assessment. In fact, only Cole and colleagues have recently reported longitudinal results of voxel‐wise brain age estimation using deep learning (Cole et al., 2017), with MAE of 4.16, 5.17 and 4.34 years for GM, WM and GM + WM modalities, respectively, and a high test–retest reliability (ICC > 0.90).

We decided to explore this aspect further by leveraging a unique dataset of 99 longitudinal MRI scans from a single, cognitively healthy volunteer acquired over a period of 17 years (aged 29–46 years). We were able to demonstrate the reliability of the patch‐wise technique (cf., Section 3.2), as well as investigate the influence of different scanner manufacturers on its accuracy (cf., Section 3.3).

## 2 MATERIAL AND METHODS

### 2.1 Training MRI dataset

**FIGURE 1:**
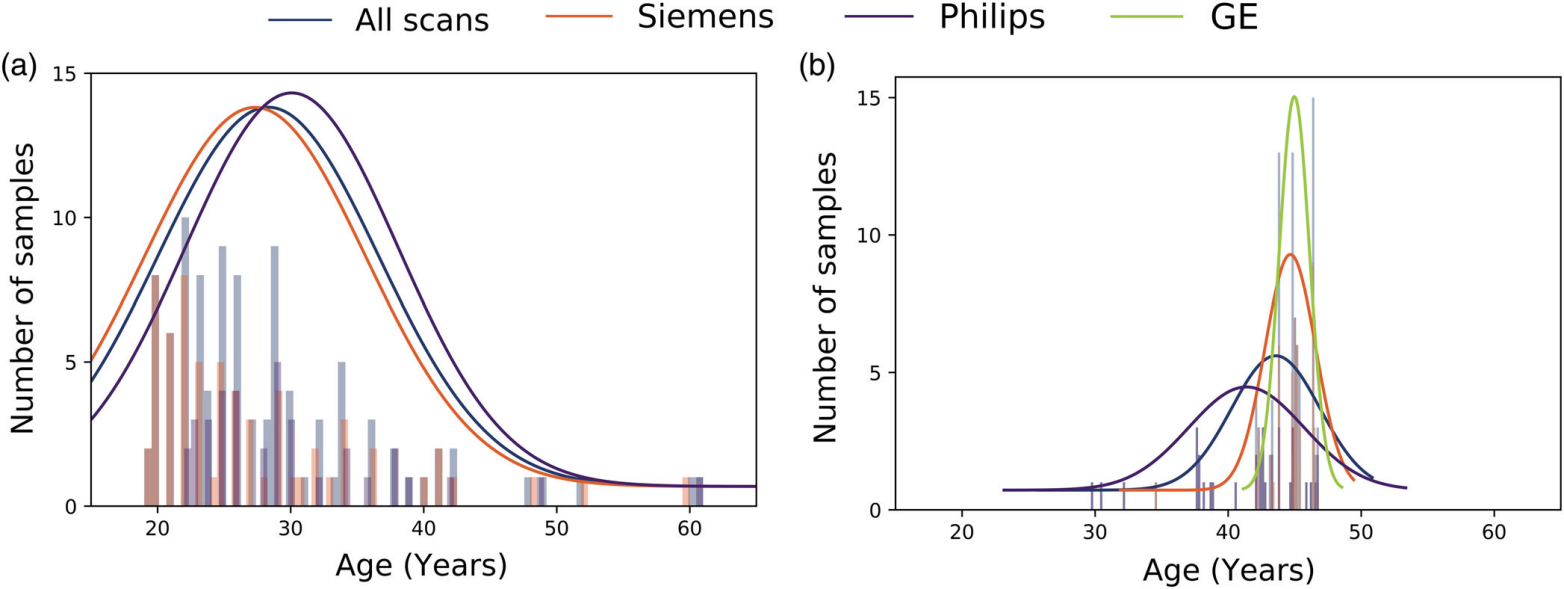
Histogram displaying the age distribution with respect to scanner manufacturer: (a) training set (*N* = 100), (b) testing set (*N* = 99)

We used the same training set from our previous study (Beheshti et al., 2019) to train the patch‐wise brain age estimation framework, specifically the MindBoggle‐101 dataset (https://mindboggle.info; Klein & Tourville, 2012), which is composed of T1w MRI scans of 100 cognitively healthy individuals between the ages of 19 and 61 years (M = 28.32, *SD* = 8.38, 44% female). This dataset was acquired on two different scanner manufacturers (Siemens, *N* = 64; Philips, *N* = 36). The age distribution of the training set is presented in Figure 1a. As described in our work, we extracted 62 cortical labels for each T1‐weighted MRI scan based on the Desikan‐Killiany‐Tourville parcellation protocol (Klein & Tourville, 2012) with the *FreeSurfer* segmentation software (http://freesurfer.net; version 5.3; default setting, recon‐all). Each brain segmentation was visually inspected thoroughly on all slices in the coronal plane. The stability of the parcellation was not measured, but given that the images came from a high number of different scanners, some notable variability was likely to occur. For example, morphometric variability was previously reported using the subset of images acquired after a scanner upgrade (Trio to Prisma) on three Siemens scanners (Potvin et al., 2019).

Next, all MRI images were mapped in pseudo‐Talairach MNI space on the basis of an affine linear registration (MINC 2.2.00 toolkit; mincresample function, default setting, and tri‐linear interpolation method), then resampled to a voxel size of 1 × 1 × 1 mm^3^. To map labels in pseudo‐Talairach MNI space, we used the mincresample function with nearest‐neighbor interpolation. The skull and other nonbrain tissues were eliminated using an intracranial mask. Finally, in order to diminish intensity variations among various scanner models, the voxel intensity of each registered MRI image was linearly mapped to a [0–100] range as follows:

(1) $${\mathrm{MRI}}_{\text{Normalized}}=100\times \frac{{\mathrm{MRI}}_{\mathrm{raw}}-\min\left({\mathrm{MRI}}_{\mathrm{raw}}\right)}{\max\left({\mathrm{MRI}}_{\mathrm{raw}}\right)-\min\left({\mathrm{MRI}}_{\mathrm{raw}}\right)}$$

Where *MRI*_raw_, min(*MRI*_raw_), and max(*MRI*_raw_) stand for the raw intensities, the minimum intensity, and the maximum intensity in each MRI image, respectively. The MRI image preprocessing was conducted using the MINC 2.2.00 toolkit.

### 2.2 Testing MRI dataset

To assess the reliability of the patch‐wise technique for longitudinal studies, we used T1w MRIs from the Single Individual volunteering for Multiple Observations across Networks (SIMON) MRI dataset (Duchesne et al., 2019). In brief, the SIMON MRI dataset is an ever‐expanding sample of convenience of longitudinal MRI scans from a cognitively healthy individual (male, ambidextrous, education: 22 years) acquired for quality control purposes in 73 sessions on 36 different scanners (13 models and three manufacturers) with different protocols, while the participant was aged 29–46 years. The SIMON dataset at the time of this study was composed of 99 T1w MRIs (Siemens Medical systems, *N* = 52; Philips Healthcare systems, *N* = 34; GE Healthcare systems, *N* = 13). Informed consent was obtained from the participant; the data is available for comparison studies (http://fcon_1000.projects.nitrc.org/indi/retro/SIMON.html). Similar preprocessing was performed on the test set. More information about this testing dataset is presented in Table 1, whilst the age distribution is shown in Figure 1b.

**TABLE 1:** Properties of testing MRI dataset

MRI manufacturers	*N*	Age
Philips	34	[29.69–46.82]; M = 41.37, *SD* = 4.40
Siemens	52	[34.52–46.82]; M = 44.64, *SD* = 1.92
GE	13	[43.27–46.41]; M = 44.97, *SD* = 1.18

Abbreviations: M, mean; SD, standard deviation.

### 2.3 Patch‐wise grading for brain age estimation

The technical details of the patch‐wise grading brain age estimation have been fully described previously (Beheshti et al., 2019). In summary, for each test label under study, a library of *N* (*N* = 20) closest subjects from the training set was composed on the basis of the sum of the squared difference criterion. It is worthwhile to mention that generating the library set was entirely independent of scanner manufacturers. Next, for each voxel *x*_*i*_ of the considered study, a patch comparison was conducted between patch *p*(*x*_*i*_) (i.e., a 7 × 7 × 7 voxel cube) with all patches *p*(*x*_*j*_) from the library set. This comparison yields the following weighting function between the voxel under study *x*_*i*_ and the voxel *x*_*j*_ from the training library (Coupe et al., 2011):

$$\mathrm{ww}\left(x_{i},x_{s,j}\right)=\left\{\begin{array}{c} e^{-\frac{{\left\|p\left(x_{i}\right)-p\left(x_{s,j}\right)\right\|}_{2}^{2}}{h\left(x_{i}\right)},\text{if}\;\mathrm{ss}>0.95}\;\\ 0,\text{otherwise} \end{array}\right)$$

(2) $$\mathrm{ss}\left(x_{i},x_{s,j}\right)=\frac{2\mu_{p\left(x_{i}\right)}\mu_{p\left(x_{s,j}\right)}}{\mu_{p\left(x_{i}\right)}^{2}+\mu_{p\left(x_{s,j}\right)}^{2}}\times \frac{2\sigma_{p\left(x_{i}\right)}\sigma_{p\left(x_{s,j}\right)}}{\sigma_{p\left(x_{i}\right)}^{2}+\sigma_{p\left(x_{s,j}\right)}^{2}}$$

In the above equation, *ww*(*x*_*i*_, *x*_*s*,*j*_) refers to the weighting function for the (*x*_*i*_, *x*_*s*,*j*_) pair; ‖.‖_2_ is the *L*^2^‐norm; and *p*(*x*_*s*,*j*_) stands for the patch which was centered on *j*^*th*^ voxel of the training sample *s* (i.e., *x*_*j*_). We carried out a preselection technique focused on the structural similarity measure criteria (Wang, Bovik, Sheikh, & Simoncelli, 2004) to pick the most insightful patches, so that we can omit weak patches that do not meet the threshold on *ss*. Finally, *μ*_*p*(*x*)_ and *σ*_*p*(*x*)_ are the mean and standard deviation of voxel values in the patch *p*(*x*), respectively, while *h* is the smoothing parameter which can be computed as follow:

(3) $$h\left(x_{i}\right)=\lambda^{2}\times {\arg\min}_{X_{s,j}}\;{\left\|p\left(x_{i}\right)-p\left(x_{s,j}\right)\right\|}_{2}+\delta$$

where, as indicated in (Coupe, Eskildsen, Manjon, Fonov, & Collins, 2012), the constants *λ* and *δ* were set to 0.5 and 10^−7^, respectively. The only difference from our prior study is that the grading value *g* at the voxel *x*_*i*_ has been modified as:

(4) $$g\left(x_{i}\right)=\frac{\sum_{s=1}^{N}\sum_{j\in V_{i}}w\left(x_{i},x_{s,j}\right).\left({\mathrm{Age}}_{\text{Test}}-{\mathrm{Age}}_{s}\right)\;}{\sum_{s=1}^{N}\sum_{j\in V_{i}}w\left(x_{i},x_{s,j}\right)\;}$$

where *V*_*i*_ refers to the search volume which ranges from 9 × 9 × 9 to 15 × 15 × 15 to discover the ideal one (Coupe et al., 2012). *Age*_Test_ and Age_s_ are the test participant's and training library subject's chronological ages, respectively.

**FIGURE 2:**
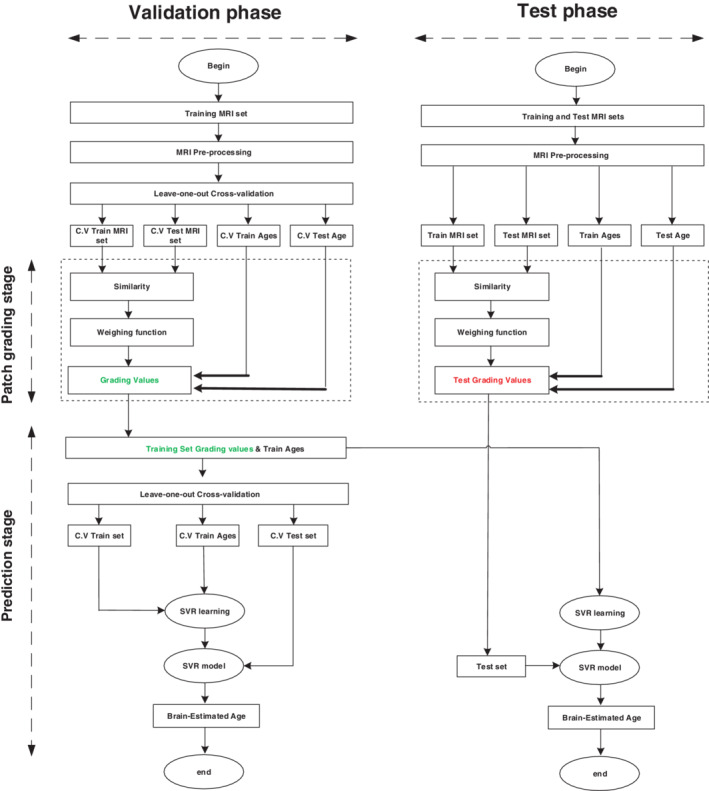
The pipeline of the proposed patch‐wise brain age framework

After computing the grading values for all voxels within a label, we calculated the final patch‐wise grading value by averaging grading values of all voxels over the respective label. Figure 2 illustrates the pipeline of the proposed patch‐wise brain age estimation framework, while the pseudo‐code of the patch‐wise grading stage is shown in Pseudo Algorithm 1.

Pseudo Algorithm 1 **The outline for the proposed patch‐wise brain age framework for each cortical label under study used in this study**

**Procedure** patch_grading (ROIs_Train_, Ages_Train_, ROI_Ttest_, Age_Test_)

N ← 20

δ ← 10^−7^

λ← 0.5

Number of voxel in ROI ← m

Select N closest subjects← computing the SSD (ROIs_Train_, ROI_Ttest_)

p(x) ← a cube of 7 × 7 × 7 voxels

**For** i = 1: m **do**

**For** s = 1: N

V_i_ ← Search volume

j ∈ V_i_

$\;\mu_{p\left(x_{i}\right)}$← mean voxel values in the patch p(xi)

$\sigma_{p\left(x_{i}\right)}$← STD voxel values in the patch p(xi)

$\mu_{p\left(x_{s,j}\right)}$← mean voxel values in the patch p(x_s,j_)

$\;\sigma_{p\left(x_{s,j}\right)}$← STD voxel values in the patch p(xi)

Ss$\left(x_{i},x_{s,j}\right)\leftarrow \frac{2\mu_{p\left(x_{i}\right)}\mu_{p\left(x_{s,j}\right)}}{\mu_{p\left(x_{i}\right)}^{2}+\mu_{p\left(x_{s,j}\right)}^{2}}\times \frac{2\sigma_{p\left(x_{i}\right)}\sigma_{p\left(x_{s,j}\right)}}{\sigma_{p\left(x_{i}\right)}^{2}+\sigma_{p\left(x_{s,j}\right)}^{2}}$ % *similarity*

$h\left(x_{i}\right)\leftarrow \lambda^{2}\times {\arg\min}_{X_{s,j}}\;{\left\|p\left(x_{i}\right)-p\left(x_{s,j}\right)\right\|}_{2}$+ δ

**For** ss **do** check

**If** ss > 0.95 **then**

$w\left(x_{i},x_{s,j}\right)\leftarrow e^{-\frac{{\left\|p\left(x_{i}\right)-p\left(x_{s,j}\right)\right\|}_{2}^{2}}{h\left(x_{i}\right)}}$ % *weighting function*

e**lse**

w(x_i_, x_s,j_) ← 0

**end if**

**end for**

$g\left(x_{i}\right)\leftarrow \frac{\sum_{s=1}^{N}\sum_{j\in V_{i}}w\left(x_{i},x_{s,j}\right).({\mathrm{Age}}_{\text{Test}}-{\mathrm{Age}}_{s)}\;}{\sum_{s=1}^{N}\sum_{j\in V_{i}}w\left(x_{i},x_{s,j}\right)\;}$ % *grading value*

**end for**

**end for**

**return** mean g(x)

**End Procedure**

### 2.4 Validation and performance assessment

To predict brain age, we used a support vector machine regression predictor implemented in MATLAB (i.e.,“fitrsvm” function, kernel: linear, KernelScale: auto). The regression was done to match the final patch‐wise average grading values in all labels to the chronological age. We assessed the prediction accuracy of the patch‐wise technique in two phases. First, we trained and validated the prediction performance of patch‐wise brain age estimation in the training set (*N* = 100) on the basis of a leave‐one‐out strategy. Second, we assessed the prediction accuracy of the patch‐wise technique by applying the patch‐wise estimation framework from the training set to the single cognitively healthy volunteer over a period of 17 years as an independent test set. The accuracy of brain age estimation was stated in terms of the mean absolute error, root mean square error (RMSE), and R‐squared. The within‐manufacturer reliability was reported based on intraclass correlation coefficient [2,1] (ICC[2,1]).

## 3 RESULTS

### 3.1 Training set model

**FIGURE 3:**
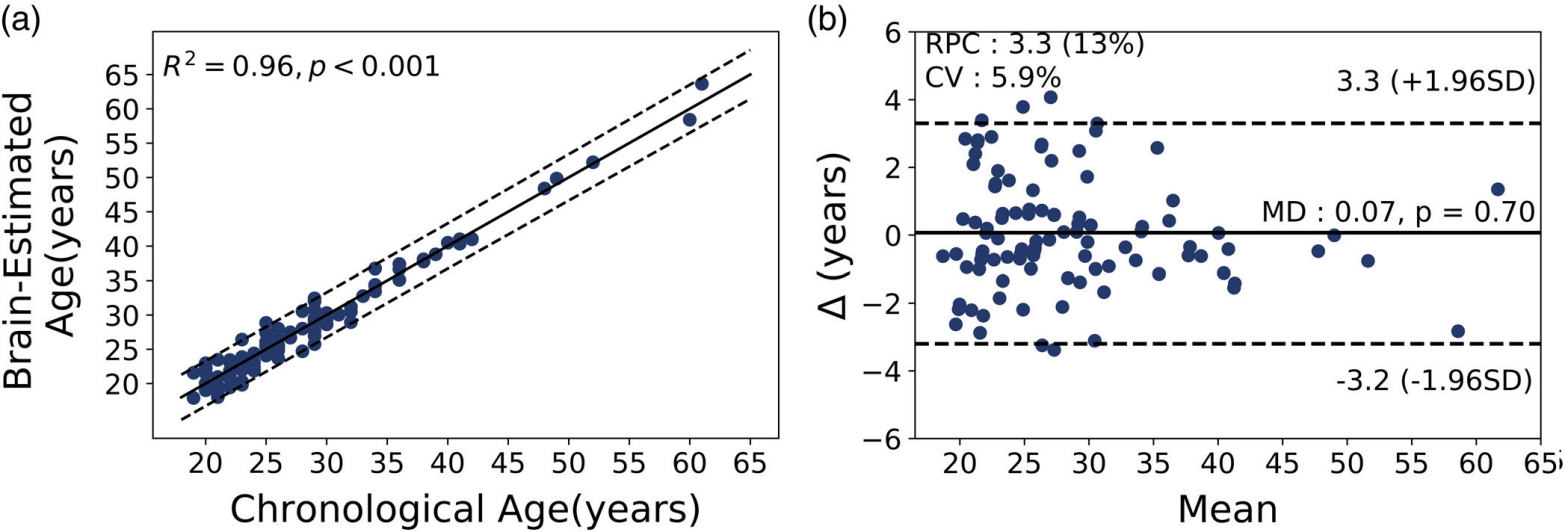
Training set model: (a) Scatter plot of estimated brain age as a function of chronological age. The solid black line shows the regression line, while the dashed black lines stand for 95% prediction band on the model prediction. (b) Bland–Altman plot between estimated brain age and chronological age. The Mean axis is the average of estimated brain age and chronological age; and the Δ axis refers to the difference between chronological age and estimated brain age. The solid black line represents the mean age difference between estimated brain age and chronological age, while the dashed black lines show ± 1.96 standard deviation. RPC and CV are reproducibility coefficient and coefficient of variation, respectively; MD is mean difference between estimated brain age and chronological age

Figure 3 shows the relationship between the estimated brain age as a function of chronological age, as well as the predicted difference (brain age delta) against the mean of chronological age and predicted brain age (i.e., Bland–Altman plot) on the training set model obtained via a leave‐one‐out strategy. Our prediction model reached a high predictive accuracy in this training set (MAE = 1.30 years, RMSE = 1.66 years and *R*^2^ = .96).

### 3.2 Longitudinal performance

**FIGURE 4:**
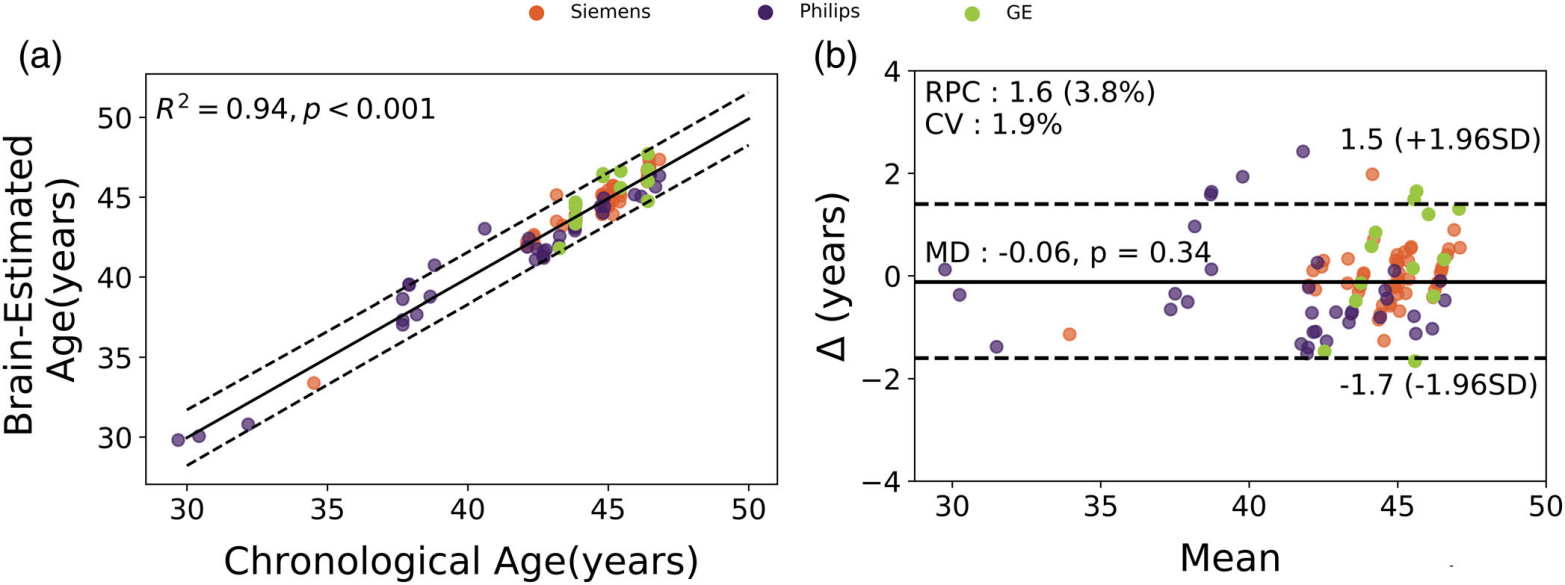
Evaluation of the performance of patch‐wise brain age on a single individual volunteer across time. (a) Scatter plot of estimated brain age as a function of chronological age. The solid black line shows the regression line, while the dashed black lines stand for 95% prediction band on the model prediction. (b) Bland–Altman plot between estimated brain age and chronological age. The Mean axis represents the average of estimated brain age and chronological age; the Δ axis refers to the difference between chronological from patch‐wise brain age. The solid black line stands for the mean age difference between estimated brain age and chronological age, while the dashed black lines show ± 1.96 standard deviation. RPC and CV are reproducibility coefficient and coefficient of variation, respectively; MD is mean difference between estimated brain age and chronological age

To assess the longitudinal reliability, we applied the brain age model from the training set to the test set. Figure 4 shows the estimated brain age plotted as a function of chronological age, as well as the predicted difference (brain age delta) against the mean of chronological age and predicted brain age (i.e., Bland–Altman plot) for a single cognitively healthy volunteer over 99 time points. The prediction accuracy on the test set was MAE = 0.63 years, RMSE = 0.80 years and *R*^2^ = .94.

### 3.3 The impact of MRI scanner manufacturers on patch‐wise results

**FIGURE 5:**
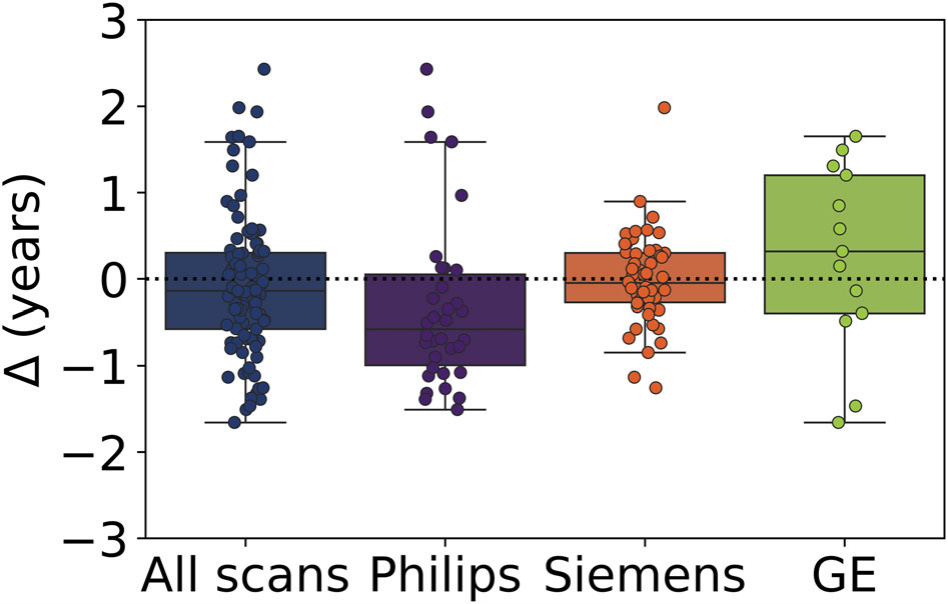
Influence of MRI manufacturer on brain age‐delta for the single individual volunteer. Δ: chronological age subtracted from the brain estimated age

The SIMON dataset allowed us to quantify the impact of the various MRI scanner manufacturers (Philips Healthcare, Best, The Netherlands; Siemens Healthcare, Erlangen, Germany; GE Medical Systems, Milwaukee, WI) on patch‐wise brain age estimation. The mean brain age delta for different MRI manufacturers were: Philips: −0.32 years; Siemens: 0.03 years; and GE: 0.23 years (Figure 5); the differences were not statistically significant (*F* = 1.82, *p* = .16; ANOVA). Within‐manufacturer reliabilities (i.e., ICC [2,1]) for Siemens, Philips and GE scanners were 0.96 [0.93, 0.97], 0.97 [0.94, 0.98], and 0.75 [0.35, 0.91], respectively.

**FIGURE 6:**
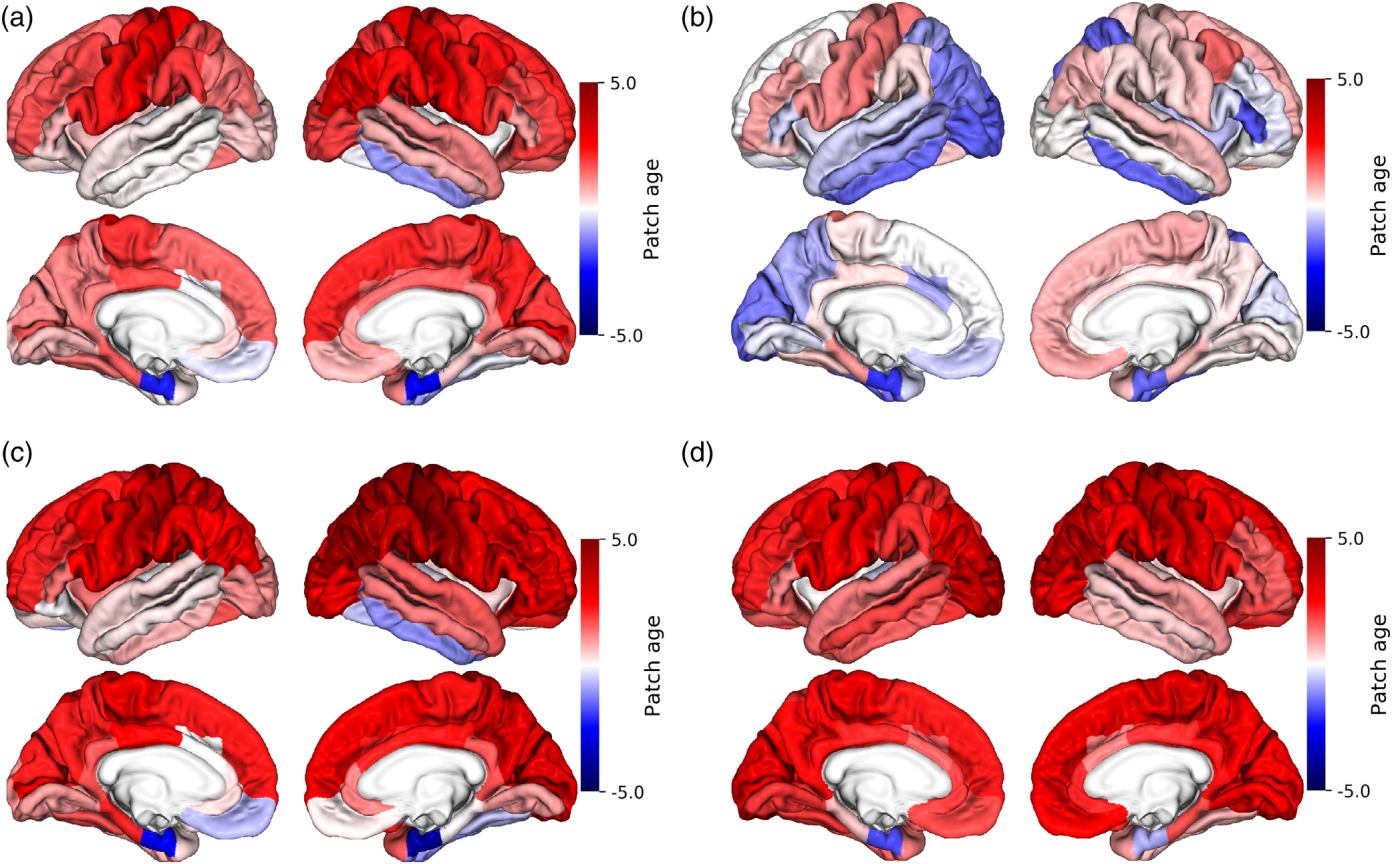
Influence of MRI manufacturer on patch‐wise grading values for the single individual volunteer at the region level. (a) All scans, (b) Philips Healthcare, (c) Siemens Healthcare, and (d) GE Medical Systems scanners

Unlike other brain age estimation techniques (i.e., voxel‐wise and region‐wise) which provide only a single scalar result, the patch‐wise technique as a corollary can show estimated grading values at the voxel and regional levels for each subject under study. Figure 6 illustrates the resulting patch‐wise grading values on our test dataset with respect to scanner manufacturer at this regional level. A summary of statistical information related to the grading values across the cortex with respect to MRI manufacturers is presented in Table 2, while Table 3 lists the highest grading values achieved from the proposed patch‐wise technique. As can be seen from Table 2, the grading value obtained from Philips scanners showed lower variation compared with Siemens and GE equipment. The MAE for the various MRI manufacturers were: Philips: 0.88 years, Siemens: 0.41 years, and GE: 0.90 years.

**TABLE 2:** The statistical information related to grading values across the cortex with respect to MRI manufacturers

	Min	Max	Mean	Standard deviation
All scans	−2.60	3.02	1.15	1.09
Philips Healthcare	−1.95	1.44	−0.05	0.79
Siemens Healthcare	−3.81	4.56	1.74	1.62
GE Medical Systems	−1.72	3.72	1.94	1.08

**TABLE 3:** Top 10 regions with highest grading values across the cortex with respect to the MRI manufacturers

All scans		Philips Healthcare		Siemens Healthcare		GE Medical Systems	
ROI	Grading value	ROI	Grading value	ROI	Grading value	ROI	Grading value
rh‐postcentral	3.02	lh‐entorhinal	−1.95	rh‐postcentral	4.56	rh‐inferiorparietal	3.72
rh‐inferiorparietal	2.98	rh‐parstriangularis	−1.62	rh‐inferiorparietal	4.36	lh‐lateraloccipital	3.57
rh‐entorhinal	2.75	lh‐lateraloccipital	−1.50	rh‐superiorparietal	4.08	rh‐postcentral	3.37
lh‐postcentral	−2.60	rh‐inferiortemporal	−1.46	rh‐pericalcarine	3.98	rh‐precentral	3.22
rh‐pericalcarine	2.43	rh‐entorhinal	−1.46	rh‐entorhinal	−3.81	rh‐paracentral	3.15
rh‐supramarginal	2.37	rh‐caudalmiddlefrontal	1.45	lh‐postcentral	3.66	lh‐cuneus	3.09
rh‐caudalmiddlefrontal	2.31	lh‐inferiortemporal	−1.44	rh‐supramarginal	3.60	lh‐parsopercularis	3.04
Lh‐entorhinal	2.27	rh‐superiorparietal	−1.44	lh‐superiorparietal	3.27	lh‐superiorfrontal	2.95
lh‐precentral	2.27	lh‐postcentral	1.32	rh‐parsopercularis	3.26	lh‐inferiorparietal	2.91
rh‐precentral	−2.20	lh‐cuneus	−1.27	rh‐precuneus	3.22	rh‐pericalcarine	2.87

*Note:* The regions were ranked based on absolute grading values.

Abbreviations: lh, Left hemisphere; rh: right hemisphere.

## 4 DISCUSSION

The main objective of this study was to assess the reliability of the patch‐wise brain age estimation technique in a longitudinal setting. In our previous study (Beheshti et al., 2019), we extended the notion of patch‐wise grading from Coupé and colleagues (Coupé et al., 2012) to estimating brain age across the cortex from 3D anatomical MRI data. Our proposed patch‐wise grading technique was tested in a cross‐sectional design and showed significantly improved prediction accuracy in an independent test set (MAE < 2 years) when compared to state‐of‐the‐art methods (Beheshti et al., 2019). When testing on the longitudinal SIMON dataset, we accurately estimated brain age with a MAE < 1 year over a long age span (17 years) covering early middle age (29–46 years old). These results support our claim that the patch‐wise technique is amenable to longitudinal brain age studies.

In a previous report (K. Franke et al., 2010), the authors investigated the influence of different scanner manufacturers on a voxel‐wise brain age estimation framework. They reported a slight difference in terms of prediction accuracy between individual scanner manufacturers. In our previous study, we assessed the influence of different MRI manufacturers on a patch‐wise technique for cross sectional studies (see Supporting Information). However, in the present study, we also explored the impact of different MRI manufacturers on the patch‐wise brain age estimation framework for longitudinal brain age estimation studies. Based on our results, we have not observed a statistically significant difference among various scanners in terms of brain age‐delta (Figure 5). Furthermore, the patch‐wise technique showed an excellent within‐manufacturer test–retest reliability for Siemens and Philips scanners (ICC > .95), well comparable with (Cole et al., 2017). As for GE, the patch‐wise technique exhibited a lower within‐manufacturer test–retest reliability (ICC ≈ .75) due to a lower sample size compared to Siemens and Philips. It would therefore suggest that the patch‐wise technique is robust, regardless of scanner manufacturers.

Regarding the patch‐grading, we expected to achieve grading values close to zero for each ROI as our longitudinal MRI scans belong to a cognitively healthy volunteer. Although creating the training library was entirely independent of scanner manufacturers, our experimental results showed similarities and differences among scanner vendors. For instance, Siemens and GE scanners showed some wide variations in terms of patch‐wise grading values in comparison to Philips (Table 2), whereas Siemens and Philips scanners exhibited similar grading values in the temporal lobe. Despite the fact that there were some differences among scanner manufacturers for patch grading, the support vector machine regression diminished these differences in prediction stage in terms of final brain age values. Therefore, there was no statistical difference among scanner manufacturers used in this study (*F* = 1.82, *p* = .16; ANOVA).

One of the strengths of this study is also an obvious limitation, namely that our test set was composed of a single individual in early middle age. In addition, the test dataset—being a sample of convenience—was not balanced with respect to scanner manufacturers. This limits the generalizability of our findings, and suggests further research using a larger test set.

## 5 CONCLUSION

This study set out to evaluate the reliability of the patch‐wise technique for longitudinal brain age estimation studies. To this end, we used a set of longitudinal MRI scans from a single cognitively healthy volunteer over a period of 17 years acquired from various scanners as an independent test set to assess the performance of the patch‐wise technique. The results confirmed that the patch‐wise technique has not only a high reliability for cross‐sectional researches, but also for future longitudinal brain age estimation studies.

## Supporting information


**Supplementary Figure 1** Influence of MRI manufacturer on brain age‐delta for an independent test set of 78 cognitively intact individuals (Beheshti et al., 2019).

## Data Availability

This study includes two samples of cognitively healthy individuals. 1‐ MindBoggle‐101 dataset (https://mindboggle.info) 2‐ the SIMON MRI dataset (http://fcon_1000.projects.nitrc.org/indi/retro/SIMON.html) Both datasets are public.
