# Supplementary material for: Patch‐wise brain age longitudinal reliability
Source: Hum Brain Mapp. 2020 Nov 18;42(3):690–8. doi: 10.1002/hbm.25253 (PMC7814761; doi:10.1002/hbm.25253)
Supplement: Supplementary file 1 — Supplementary Figure 1 Influence of MRI manufacturer on brain age‐delta for an independent test set of 78 cognitively intact individuals (Beheshti et al., 2019). [file HBM-42-690-s001.docx]

Supplementary Information

**The impact of MRI scanner manufacturers on patch-wise results for cross sectional studies.**

**Material and methods**

To show the impact of MRI scanner manufacturers on patch-wise results for cross sectional studies, we used the prediction results from our previous study (Beheshti et al. 2019). Briefly, the training set was consisted from 100 cognitively unimpaired adults from 100 cognitively healthy individuals from the MindBoggle dataset (M = 28.32, SD = 8.38, 44% female, aged 19–61 years) (same training set with the present study) and independent test set was involved of 78 individuals (M = 40.88, SD =12.13, aged: 21–60, 51% female, scanner: 52 Philips, 13 Siemens, 13 GE).

The test data set was collected from multisite datasets (i.e., Functional Biomedical Informatics Research Network (FBIRN), Information eXtraction from Images (IXI), F.M. Kirby Research Center neuroimaging reproducibility data (KIRBY-21), International Consortium for Brain Mapping (ICBM), Open access series of imaging studies (OASIS)).

**Results:**

The mean brain age delta for different MRI manufacturers were Philips -0.64 years, Siemens -1.27 years, and GE -1.45 years; the differences were not statistically significant (F = 0.58, p = 0.58; ANOVA).

| 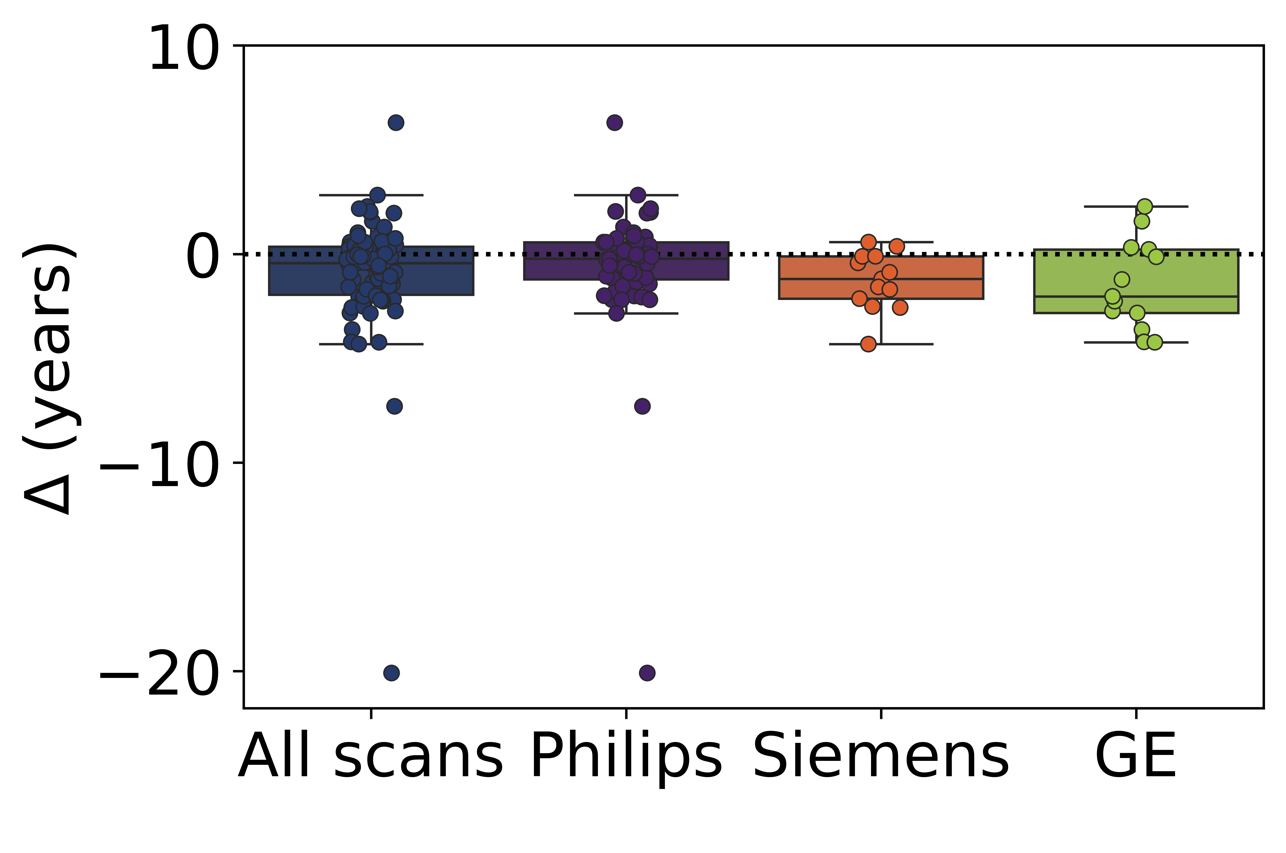 |
| --- |
| **Supplementary Figure 1.** Influence of MRI manufacturer on brain age-delta for an independent test set of 78 cognitively intact individuals (Beheshti et al. 2019). |
